# Understanding the role of aliovalent cation substitution on the li-ion diffusion mechanism in Li_6+*x*_P_1−*x*_Si_*x*_S_5_Br argyrodites†

**DOI:** 10.1039/d3ma01042b

**Published:** 2024-01-15

**Authors:** Tammo K. Schwietert, Ajay Gautam, Anastasia K. Lavrinenko, David Drost, Theodosios Famprikis, Marnix Wagemaker, Alexandros Vasileiadis

**Affiliations:** a Storage of Electrochemical Energy, Department of Radiation Science and Technology, Faculty of Applied Sciences, Delft University of Technology Mekelweg 15 2929JB Delft The Netherlands m.wagemaker@tudelft.nl a.vasileiadis@tudelft.nl

## Abstract

Due to their high ionic conductivity, lithium-ion conducting argyrodites show promise as solid electrolytes for solid-state batteries. Aliovalent substitution is an effective technique to enhance the transport properties of Li_6_PS_5_Br, where aliovalent Si substitution triples ionic conductivity. However, the origin of this experimentally observed increase is not fully understood. Our density functional theory (DFT) study reveals that Si^4+^ substitution increases Li diffusion by activating Li occupancy in the T4 sites. Redistribution of Li-ions within the lattice results in a more uniform distribution of Li around the T4 and neighboring T5 sites, flattening the energy landscape for diffusion. Since the T4 site is positioned in the intercage jump pathway, an increase in the intercage jump rate is found, which is directly related to the macroscopic diffusion and bulk conductivity. Analysis of neutron diffraction experiments confirms partial T4 site occupancy, in agreement with the computational findings. Understanding the aliovalent substitution effect on interstitials is crucial for improving solid electrolyte ionic conductivity and advancing solid-state battery performance.

## Abstract 2

The Si-induced uniform Li distribution enhances intercage diffusion, enabled through the T4 interstitial positions, resulting in increased macroscopic diffusion and ionic conductivity.

## Graphical abstract

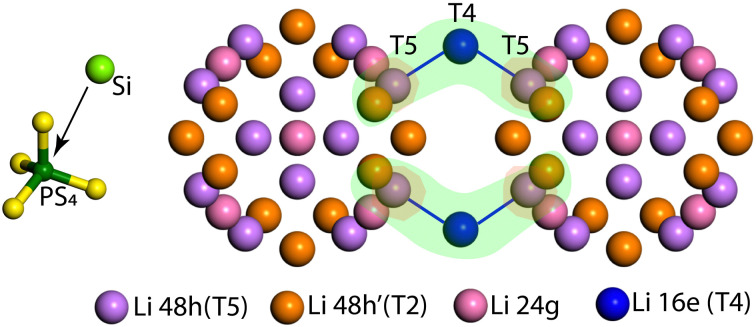

## Introduction

All-solid-state batteries present a promising solution to the safety risks associated with volatile liquid electrolytes. Moreover, these batteries offer the potential for higher energy densities by enabling the use of Li metal and Si as an anode.^1,2^ One of the main obstacles to enabling solid-state batteries is the restricted power density originating from the poor Li-ion diffusion at the interface with the electrode. Several solid electrolytes have been developed, including oxides, halides, borohydrides, and sulfides. Among them, sulfide-based solid electrolytes are promising due to exceptionally high ionic conductivity and low grain boundary resistance.^1–3^ Recently, the argyrodite family Li_6_PS_5_X (X = Cl, Br, and I) has attracted considerable interest due to its high ionic conductivity (1–10 mS cm^−1^ at room temperature^4,5^) and the relatively low grain-boundary resistance because of their mechanically soft nature. However, the electrochemical instability at low and high potentials, especially in the vicinity of the negative and positive electrodes, remains a significant challenge.^3,6–8^

Fig. S1 (ESI†) shows the (ordered) crystal structure of argyrodite (Li_6_PS_5_X, X = Cl, Br, or I), in the cubic *F*4̄3*m* space group. Halide ions occupy the face-centered cubic lattice on the tetrahedral Wyckoff 4a sites, and S^2−^ (not bonded to P) occupies the tetrahedral sites (Wyckoff 4c). In the cubic phase of the argyrodite, the anion framework forms 136 interstitial tetrahedral voids in the unit cell, which are suitable for cation occupancy. Four of these voids are filled by P^5+^ cations at the Wyckoff 4b site, forming PS_4_^3−^ tetrahedra. The remaining 132 tetrahedral voids can accommodate lithium and are classifiable based on the number of S-ions (16e) they share (either through face, edge, or corner) with PS_4_^3−^ tetrahedra. Accordingly, the tetrahedral site can be divided into five distinct types (Types 1–5). Type 1 (T1) sites share faces, and Type 2 (T2) tetrahedra share edges with PS_4_^3−^ tetrahedra. On the other hand, Type 3 (T3) and Type 4 (T4) tetrahedra share four and three corners with PS_4_^3−^ tetrahedra, respectively. Type 5 sites share two corners with PS_4_^3−^ tetrahedra, while Type 5a is situated at the shared face of two neighboring T5 tetrahedra, resulting in a trigonal bipyramidal environment due to their close proximity. T5, T2, and T5a Li sites form cage-like clusters around the 4d site. The T4 site (Wyckoff 16e) is of particular interest as it is located between two cages, which is pivotal in improving the long-range lithium diffusion in Li-argyrodites.

Various approaches have been successfully implemented to improve the ionic conductivity of Li-argyrodites. Altering the ratio of halogen anions (Li_6−*x*_PS_5−*x*_(Cl,Br,I)_1+*x*_) increases the number of vacancies^9,10^ while tailoring the exchange between S^2−^/X^−^ (X = Cl, Br, and I) anions on the Wyckoff 4c site, referred to as a “site-disorder”, makes Li pathways more interconnected.^11^ Another practical approach to increase the ionic conductivity of Li-argyrodites is substitutions with aliovalent cations such as Si^4+^(*r*_Si_^4+^ = 26 pm), Ge^4+^(*r*_Ge_^4+^ = 39 pm), Sn^4+^(*r*_Sn_^4+^ = 55 pm) and Al^3+^(*r*_Al_^3+^ = 39 pm).^12–14^ These substitutions increase the lithium-ion concentration as well as the lattice volume due to their higher ionic radius compared to P^5+^(*r*_P_^5+^ = 17 pm). Substitutions can also effectively alter other relevant electrolyte properties such as electrochemical stability,^15^ air stability,^16^ elasticity,^17^ hardness,^17^ and fracture toughness.^17^ Kraft *et al.* recently demonstrated that Ge substitution (Li_6.6_P_0.4_Ge_0.6_S_5_I) enables extremely high conductivity (18.4 mS cm^−1^) after sintering.^12^ Further, Zhou *et al.* showed that Si and Ge substitution in thioantimonate argyrodites Li_6+*x*_Sb_1−*x*_M_*x*_S_5_I (M = Si and Ge) practically increases the ionic conductivity up to 24 mS cm^−1^ for sintered pellets.^18^ Si^4+^ is shown to be an effective substituent in sulfide-based electrolytes with the Li_9.54_Si_1.74_P_1.44_S_11.7_Cl_0.3_ composition in the LGPS structure delivering one of the highest conductivities reported for solid electrolytes in general (25 mS cm^−1^).^19^

Similarly, Si^4+^ substitution benefits the kinetics in the Li-argyrodite Li_6_PS_5_Br structure. Minafra *et al.* reported a three-fold increase in Li-ion conductivity upon Si^4+^ substitution, which cannot be explained solely by the increase in charge carrier concentration (8.3% for Li_6.5_P_0.5_Si_0.5_S_5_Br)^13^ A more detailed diffusion mechanism is needed to understand this effect, rooted in the hopping mechanisms Li-ions follow to move through the argyrodites crystal lattice. While the T5 site is assumed to be the single active Li site,^9^ recent studies propose multiple additional sites contributing to the diffusion mechanism. For instance, the T4 site has been identified as a potential interstitial site that can stimulate diffusion by flattening the energy landscape. This has been demonstrated in a similar structure to Li-argyrodite, Li_6.6_Al_0.15_Si_1.35_S_5.4_O_0.6_. However, a complete mechanism for all contributing sites has not yet been presented.^20^ Hogrefe *et al.* also observed that in the case of Ge-substituted samples (Li_6+*x*_P_1−*x*_Ge_*x*_S_5_I), small lithium occupancies exist in the newly explored positions T2 and T4. This phenomenon facilitates long-range lithium transport by activating a diverse range of jump processes.^21^ However, additional research is needed to comprehensively understand the hopping mechanism within the argyrodite structure and how it affects the ion dynamics and stability of the material. Further investigations in this area will contribute to a deeper understanding of the material's behavior and the discovery of new structures for enhancing ionic conductivity and other desirable properties.

In this work, we aim to understand the effect of aliovalent Si^4+^ substitution on the transport properties of Li_6_PS_5_Br. It is important to highlight that the experimental solubility limit of Si^4+^ in Li_6_PS_5_Br indicated a silicon content limit of *x* = 0.3.^13^ However, our study intentionally employed silicon content levels beyond this limit to gain a more comprehensive understanding of the effects of aliovalent substitution on ionic transport. We perform an *ab initio* molecular dynamics (AIMD) study on the Li_6+*x*_P_1−*x*_Si_*x*_S_5_Br structure, revealing the stabilization of the T4 site, which is enabled by the excess Li inserted in the structure. We report increased conductivity for higher substitution concentrations correlated to the partial occupancy on the T4 site, facilitating an energetically more facile route for intercage diffusion. Moreover, we confirm the occupancy of the T4 site by Rietveld refinement of neutron diffraction, validating the calculations and providing an enhanced fundamental understanding of structure-transport correlations in Li-argyrodites altered by cation substitution.

## Methods

### *Ab initio* molecular dynamics (AIMD)

AIMD simulations using the Vienna *Ab Initio* Simulation Package (VASP) are performed to evaluate the Li-ion diffusion in argyrodites. The simulations use the generalized gradient approximation (GGA) and the PAW-PBE basis set. The argyrodite structure is taken from literature,^9^ and the Si^4+^ atoms are substituted on the P^5+^ sites according to the literature.^13^ Li-ions are added on the T5 sites furthest away from neighboring Li-ions to minimize Coulombic interactions.^9^ Structure optimization resulted in lattice parameters converging within 1% of the experimentally determined ones. For the AIMD simulations, the cutoff energy was set at 350 eV, and the *k*-points mesh was reduced from 4 × 4 × 4 to 1 × 1 × 1.^9^ Long AIMD simulations of 800 ps occurred in the argyrodite unit cell to study the diffusion behavior in five Si concentration steps (*x* = 0, *x* = 0.25, *x* = 0.5, *x* = 0.75, *x* = 1 in Li_6+*x*_P_1−*x*_Si_*x*_S_5_Br) in the temperature range 600–850 K. Shorter simulations of 100 ps at 600 K were performed in a 2 × 1 × 1 argyrodite supercell to determine the occupation behavior and jump analysis, offering a denser Si concentration step (*x* = 0, *x* = 0.125, *x* = 0.25, *x* = 0.375, *x* = 0.5, *x* = 0.625, *x* = 0.75, *x* = 0.875, *x* = 1 in Li_6+*x*_P_1−*x*_Si_*x*_S_5_Br). The timestep was set to 2 fs for all simulations. The AIMD simulation is then separated into 10 parts for which the mean diffusion constant and standard errors are calculated.^22^ To determine the Li-ion conductivity in Li-argyrodites, first, the tracer diffusivity *D* is calculated by eqn (1),1
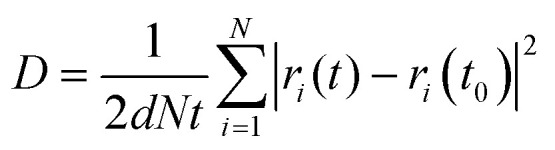
where *N* is the total number of diffusing atoms, *r*_*i*_(*t*) is the position vector of each Li atom *i* in the simulation, *d* is the number of diffusion dimensions (here 3), and *t* is the simulation time. From the diffusion constant, the conductivity can be calculated using the Nernst–Einstein relation:
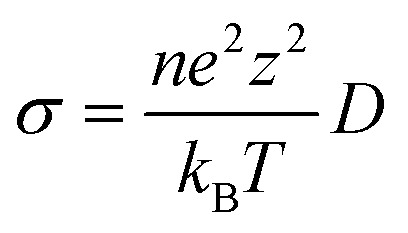
where *e* is the electron charge, *n* is the particle density, *z* is the charge of the diffusive element, and *k*_B_ is the Boltzmann constant.

To evaluate jump rates in the MD simulations, jumps between specific sites in the structure are monitored. The definition of a jump in the simulation includes assuming a radius around the site position, and if a Li atom jumps from within the site radius to another site radius, a jump is counted. The monitoring and analysis of individual sites are based on our previously developed computational tools,^9^ which involve the assignment of a spherical radius around each crystallographic position and tracking occupation and hopping behavior per site type. The T5, T4, and T2 interstitial positions are in close proximity (1.2 and 1.8 Å for T5-T2 and T5-T4, respectively) in the crystal lattice, complicating the analysis when probing them simultaneously as the defined spherical sites have to be smaller to avoid overlap, leading to capturing less than 60% of the Li density. To account for this, we also probed the system by defining all sites individually, capturing almost 100% of Li density, and ensuring the consistency of the results. This analysis is presented in ESI† C, followed by a comparison with experimental data. We perform radial distribution function (RDF) analyses by considering the T5 (48 h) site and calculating distances only during T5–T5 lithium transitions, aiming to capture the difference in the energy landscape upon Li intercage motion and differentiate it from the background arising from total RDF calculations that make the T4 detail indistinguishable due to its low occupancy.

### Neutron powder diffraction

To identify the possible interstitial sites of lithium occupancies, we re-analysed neutron diffraction data of Si substitution from Minafra *et al.*^13^ and pristine Li_6_PS_5_Br neutron diffraction data from Gautam *et al.*^11^ The measurement of both samples were performed at room temperature. High-resolution neutron powder diffraction data of Si substitution in Li_6_PS_5_Br materials were collected at the Heinz Maier-Leibnitz Zentrum (research reactor FRM II, Garching b. München, Germany) using the high-resolution diffractometer SPODI^11,13^ and monochromatic neutrons (wavelength = 1.54817(2) Å). The strategy of data collection and experimental parameters were shown in Li_6+*x*_P_1−*x*_Si_*x*_S_5_Br.^11,13^

### Rietveld refinement

The TOPAS software tool was used to perform Rietveld refinements of neutron diffraction data. The quality of the fits was determined using the goodness-of-fit (GOF) indicator and *R*_wp_. The following parameters were refined: (1) 10 coefficients for a Chebyshev function were used to fit the background and peak shape modeled by the modified Thomson-Cox-Hastings pseudo-Voigt function; (2) scale factor, lattice parameter, and zero error; (3) isotropic atomic displacement parameter; and (4) atomic occupancies of the free S^2−^ (Wyckoff 4d) and Br^−^ (Wyckoff 4a) anions (refined since these two anions can be exchanged), resulting in site-disorder (Br^−^/S^2−^). The constraint on the Wyckoff 4a site (occupancies of Br^−^(4a) + S^2−^(4a) = 1) and the Wyckoff 4d site is equal to 1 (occupancies of S^2−^(4d) + Br^−^(4d) = 1) due to charge balance in the structure. Additionally, Si occupancies are also refined. The stability of the refinements was ensured by allowing the refinement of multiple correlated parameters simultaneously. Finally, lithium occupancies on the possible interstitial sites were investigated. Upon refining over several cycles. Finally, the reported structural models were obtained, allowing for the refinement of all these structural parameters simultaneously.

## Results

**Fig. 1:**
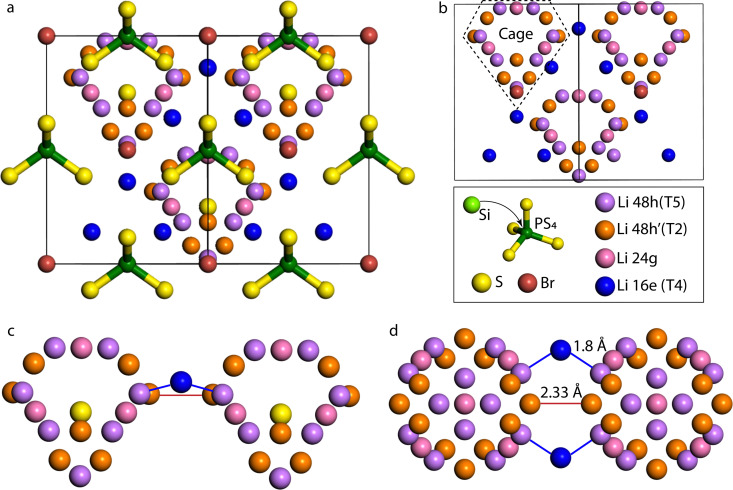
(a) The cubic structure of Li_6_PS_5_Br with the T5 lithium sites (Wyckoff 48h) in purple, T2 lithium sites (Wyckoff 48h′) in orange, the T4 lithium sites (Wyckoff 16e) in blue, the Si^4+^/P^5+^ sites in lime/green, the Wyckoff 4a and 4c sites are occupied by Br^−^ (brown colour) and S^2−^ (yellow colour), respectively. (b) Lithium forms cage-like substructures around the Wyckoff 4c site; the T4 sites are located between the cages, connecting the cages. (c) side view and (d) top view of two Li diffusion cages, depicting two intercage diffusion paths that determine the macroscopic diffusion, the Li jump pathway *via* T2–T2 sites, and the T5–T4–T5 jump pathway.

Lithium argyrodite crystallizes in the *F*4̄3*m* spacegroup, the structure of which, along with a focus on the Li sublattice and the possible jumps, are shown in Fig. 1. In the argyrodite structure, Li-ions jump between unoccupied sites through the lattice. Considering Li resides on the T5 sites, three different jumps are distinguished: doublet, intracage, and intercage.^9^ Doublet jumps occur between paired T5 sites *via* T5a (24g) (distance 1.9 Å), and intracage jumps occur between pairs of T5 sites (distance 2.25 Å) within a cage. The intercage jumps, essential for long-range lithium-ion diffusion, occur between the cages *via* T2–T2 and T5–T4–T5 pathways;^9^ the two paths are shown in Fig. 1c and d. As lithium moves fast in the argyrodite structure, partial occupancies on intermediate and meta-stable sites are difficult to assign as the probability density of Li spreads out over multiple sites. In recent literature, high-resolution neutron and X-ray diffraction experiments have shown partial occupancies on two additional Li sites between the T5 positions: T2 site^11,23,24^ and the T5a site (Wyckoff 24g).^11,13,25^ In our simulations, there is no clear indication of Li occupation on the T5a site (<0.05 occupancy). Additionally, several other vacant Li sites in the lattice can be occupied,^24^ where the T4 site (Wyckoff 16e) is of particular interest as it is located between two cages. It has been theorized that if the T4 site was occupied, diffusion through this site could improve the macroscopic diffusion as it lowers the energy barrier for intercage jumps.^20,23,26^

**Fig. 2:**
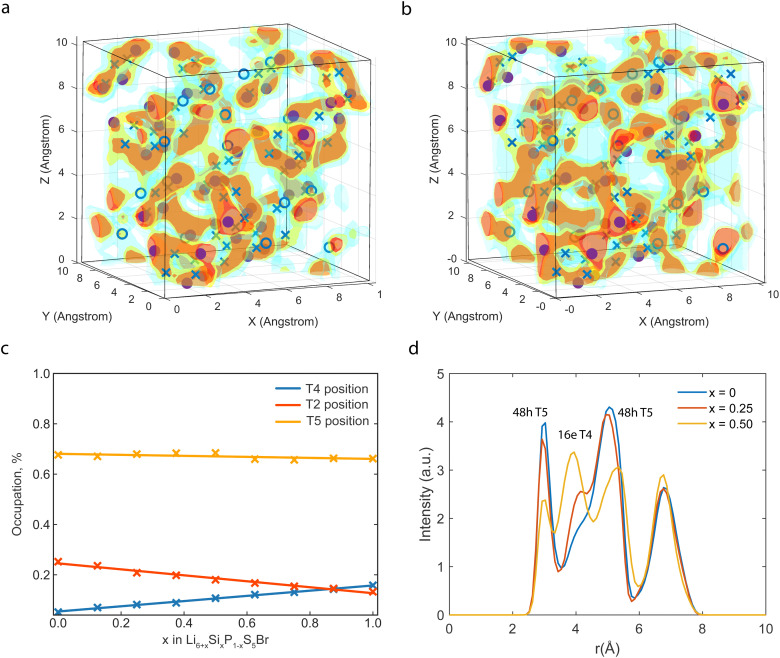
(a) Density plot of Li for the Li_6_PS_5_Br and (b) the fully substituted Li_7_SiS_5_Br during a 100 ps 600 K AIMD simulation. The solid spheres indicate the T5 positions, the crosses indicate the T2 positions, and the circles indicate the T4 positions. The red color indicates a high lithium density. With increasing Si, the intensity of the T4 position increases. (c) Percentage of Li that occupies the T4, T2, and T5 positions in a 600 K AIMD simulation. (d) Radial distribution plot between Li and Si^4+^/P^5+^ measured during T5–T5 transitions, the peaks at 3 Å, 5 Å, and 7 Å correspond to the T5 position, and with increased substitution, a peak at 4 Å is found, which corresponds to the T4 position.

To evaluate the effect of the Si substitution in Li-argyrodite, we increase the substitution concentration *x* in Li_6+*x*_P_1−*x*_Si_*x*_S_5_Br from *x* = 0 to *x* = 1 in eight concentration steps. As experimental research predicts that Si^4+^ cation replaces P^5+^ cation on the Wyckoff 4b positions,^13^ Si^4+^ is correspondingly homogeneously (maximizing distances) substituted on the P positions in the DFT simulations. The relaxed structures show a linear relationship between lattice parameters and doping concentration as follows Vegard's law due to a higher ionic radius of Si^4+^(*r*_Si_^4+^ = 26 pm) compared to P^5+^(*r*_P_^5+^ = 17 pm), and is in good agreement with the literature (Fig. S2, ESI†).^13^ To visualize Li migration, the pristine (P-based) and fully substituted (Si-based) argyrodite Li densities are shown in Fig. 2a and b. Lithium density plots from Li_6_PS_5_Br show high lithium densities on the T5 sites, where the cage-like structures in which lithium diffuses are clearly visible. The relatively high lithium concentration between T5 sites is assigned to the T2 position in both the P-based and Si-based structures. Additionally, for the Si-based structure, a sharp increase in Li density between the Li cages is found, and analyzing these positions in the *F*4̄3*m* spacegroup indicates the occupation of the T4 position. The T4 position is enabled by the lower valence of Si^4+^ which provides a Li excess in the Li_7_PS_5_Br argyrodite structure, because of charge balance. These additional Li^+^ ions in the structure occupy the lowest energy vacant site and can also be influenced by the difference in valence between Si^4+^ and P^5+^, which have a reduced repelling Coulombic force on Li^+^ atoms changing the local energy landscape around the Si atoms.

The distribution of Li ions among the T4, T2, and T5 sites is determined by analyzing the time fraction that Li resides on a specific site in the AIMD simulation, shown in Fig. 2c. Fig. 2c demonstrates a noticeable increase in the T4 occupancy as the Si^4+^ content increases in the structure and agrees with the higher Li densities at this site in Fig. 2a and b. Following Fig. 2c, the percentage of Li at the T5 site remains relatively constant, whereas the occupancy of the T2 site decreases despite the overall increase in the total lithium content within the structure. Hence, Si doping induces a redistribution of Li^+^ ions within the material. Specifically, Li tends to occupy the T2 position less frequently, and the excess Li introduced by Si doping is distributed between the T4 and T5 positions.

Fig. 2d depicts the Li-(P/Si) RDF measured during T5–T5 transitions, aiming to investigate intercage Li-ion motion *via* the T4 position. The RDF shows the integrated Li density over the simulation time as a function of the distance to the nearest 4b site. Two peaks around 3 Å and 5 Å corresponding to two T5 positions are visible for the P-based argyrodite. Si substitution results in an extra peak of around 4.2 Å, coinciding with the distance from the Si^4+^ or P^5+^ positions to the T4 Li positions. Hence, the RDF shows that the diffusing ions occupy the T4 positions when Si^4+^ is increasingly substituted into the unit cell. In the RDF, a redistribution of the Li density for the diffusion pathway is shown as the Li density is more spread out over the diffusion path, and the peaks corresponding to the T5 sites are lower. The spreading of the Li density indicates that Li is less constrained to the specific site positions, which could correlate to the flattening of the energy landscape and, hence, a more facile jump pathway.^26^ This flattening is caused by the partial Li occupation on the T4 positions that, due to Coulombic interactions in the lattice, distributes Li more uniformly around the T4 and two adjoining T5 sites.

**Fig. 3:**
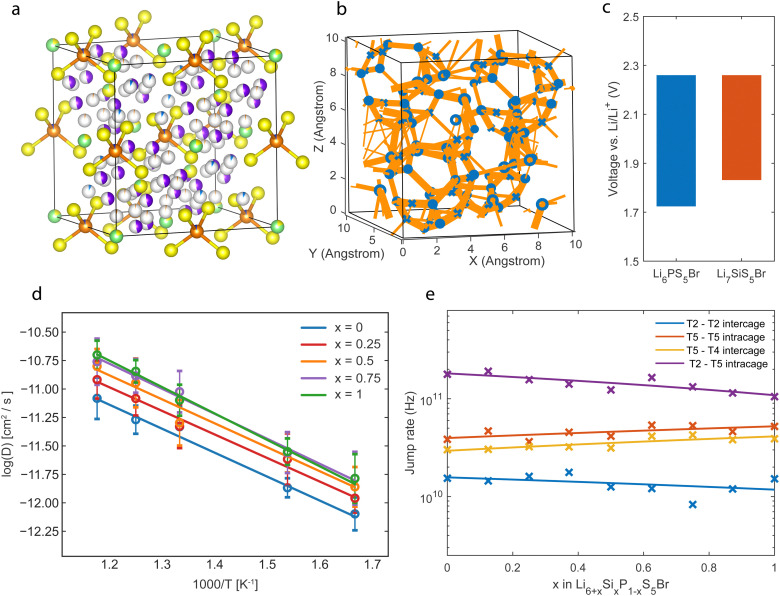
(a) Structure of Li_6.125_P_0.875_Si_0.125_S_5_Br with fractional occupancies based on neutron diffraction data analysis.^13^ Yellow represents sulfur, green is the bromide, and orange is the phosphorous. The T5 is depicted in purple, the T2 in red, and the T4 in blue. (b) Jump statistics plot of Li_6.5_P_0.5_Si_0.5_S_5_Br, thicker lines correspond to a higher jump rate between positions. (c) Thermodynamic stability window of Li_6_PS_5_Br and Li_7_SiS_5_Br, based on decomposition to products in the Materials Project database.^7,27^ (d) Diffusivity *versus* temperature plot of Li_6+*x*_P_1−*x*_Si_*x*_S_5_Br for different *x*. (e) Jump rate of different site jumps for Li_6+*x*_P_1−*x*_Si_*x*_S_5_Br in a 600 K AIMD simulation.

Further, we analyze the Li site occupancies in the Si-substituted argyrodite structures by Rietveld refinement of neutron diffraction data of Li_6.125_P_0.875_Si_0.125_S_5_Br composition. The site occupancies obtained by a Rietveld refinement are shown in Fig. 3a, and the fitted parameters of the Rietveld refinement are shown in Table S1 (ESI†). A normalized fractional occupancy of 5.03% is experimentally found in the T4 position, consistent with the value of 6.98% derived from the MD simulations at 600 K for the same Si concentration (Table S2 and Fig. S5, ESI†), confirming that the T4 position is partially occupied in the substituted structures. The relative ratios of the T2 and T5 sites are different compared to the MD simulations, where a relatively low occupation of T2 occupancy (4.90%) is found in experiments. This discrepancy can be explained by the proximity of the T2 site to the T5 site, which is difficult to disentangle as the radii that determine these sites are relatively close. Li moving from the T2 site to the T5 site overestimates the occupancy on the T2 site; however, the total occupancy on these positions remains unchanged, and the T2–T5 local intracage exchange does not affect the analysis of the macroscopic kinetic mechanism.

The jump statistics captured during the MD simulation for the Li_6.5_P_0.5_Si_0.5_S_5_Br composition are visualized in Fig. 3b, showing clear connections through the T4 positions. Fig. 3c shows the electrochemical stability of the fully substituted Li_7_SiS_5_Br and Li_6_PS_5_Br structures. This electrochemical stability window is evaluated by the formation energy towards the most favorable decomposition products obtained from the Materials Project database.^7,27^ The oxidation potential is equal for both phases as it is determined by the sulfur (S^2−^/S^0^) redox. The reduction potential that is affected by the phosphorus (P^5+^/P^0^) and silicon redox (Si^4+^/Si^0^) shows a higher reduction potential for increasing substitution. The lower reduction potential of Si compared to P can rationalize this, making the decomposition reaction towards reduced Si phases energetically more favorable over reduced P-based decomposition products, narrowing the electrochemical stability for Si-substituted argyrodites.

We then evaluate the diffusivity of Li-ions in the Si-substituted argyrodites by constructing Arrhenius plots shown in Fig. 3d. Simulations put forward an increasing trend in diffusivity and conductivity with higher Si concentrations, in agreement with the trend observed from direct experimental impedance spectroscopy results.^13^ However, quantitatively, AIMD simulations overestimate the tracer conductivities compared to experimental impedance values (64 mS m^−1^*vs.* 2.4 mS cm^−1^, Li_6.5_P_0.5_Si_0.5_S_5_Br). This overestimation could be due to the nature of AIMD simulations, which probe systems with limited size and for short timeframes^28^ and assume perfect crystals without any contributions from grain boundary resistance and contact losses.^9^ Further, it has been demonstrated that several solid-state material families suffer from quasi-linear non-Arrhenius regimes present at lower temperatures that are less accessible with AIMD simulations, introducing an overestimation when extrapolating from higher temperatures.^29^ The experimental activation energies found in the literature (∼0.2 eV^13^) agree with those found in the AIMD simulations (∼0.19 eV), shown in Fig. S3 (ESI†).

The jump-type influence on the increase in Li conductivity is also investigated. The jump rates between different Li sites in the Li_6+*x*_P_1−*x*_Si_*x*_S_5_Br structure are calculated and shown in Fig. 3e. The T2–T5 jump path, corresponding to the jumps inside the cages, shows the highest jump rates, which coincide with the large delocalization of the Li density in Fig. 2a between the T2 and T5 sites, indicating fast Li movement between the two positions.

To evaluate the jump statistics between the Li cages (intercage) jumps, the T5–T4–T5 jumps and T2–T2 jumps are considered, as shown in Fig. 1d. The jump frequency of the T5–T4–T5 path is significantly higher than the T2–T2 jump path and thus has a more significant contribution to the macroscopic diffusion. This shows that the intercage diffusion predominantly moves through the T4 position compared to the T2–T2 jump pathway. Additionally, an increase in the T4–T5 jump rate as a function of Si concentration is observed. This increase is consistent with Fig. 2d, where the Li density for the jump pathway is flattened at higher Si substitution, indicating faster diffusion. Because the intercage jump rate is the bottleneck for macroscopic diffusion, the faster jump rate through the T4 site directly influences the solid electrolyte's total macroscopic diffusivity and ionic conductivity, as shown in Fig. 3d.

Finally, a correlated increase in jump rate for the T5–T4 and T5–T5 jumps with increasing Si substitution is shown in Fig. 3e, and these jumps correspond to the inter-cage and intra-cage jumps, respectively. The correlation suggests that a jump from the T4 to a T5 site promotes a jump between two neighboring T5 sites. The above indicates a correlated interstitially driven mechanism, where Li on the T4 interstitial site pushes a Li on the T5 position to a neighboring T5 position. This mechanism benefits macroscopic diffusion as it improves inter- and intracage diffusion. A decrease in the T5–T2 jump rate compromises this correlated increase. However, it does not affect the macroscopic diffusion as this jump rate is not a limiting factor for the macroscopic diffusion.

The presence of such correlated lithium transport indicates frustration, a phenomenon known for flattening the energy landscape in superionic conductors.^30–33^ Various frustration mechanisms have been explored to understand the high diffusivity observed in materials such as Li_6_PS_5_X argyrodites,^24^ garnets,^34^ Li_3_MX_6_-type halides,^35^ thiophosphates,^36^ nanostructured Ba_1−*x*_Ca_*x*_F_2_,^33^ and others. In the case of Li_6+*x*_P_1−*x*_Si_*x*_S_5_Br, silicon substitution induces geometric frustration by mixing small P^5+^(*r*_P_^5+^ = 17 pm) and large Si^4+^(*r*_Si_^4+^ = 26 pm) cations. The resulting steric and Coulombic interactions force lithium to occupy more T4 positions, approximately 4.2 Å away from Si^4+^/P^5+^ positions, rather than T5 positions at the closer distance of 3 Å (as depicted in RDF, Fig. 2d). This occupation of higher energy sites enables disordering of the diffusing atom sublattice and energy landscape, establishing a correlated hopping mechanism.^37^ Hence, the geometric frustration, arising from optimizing Coulombic interatomic interactions and accommodating differing cationic radii contributes to the enhanced ionic conductivity observed in Li_6+*x*_P_1−*x*_Si_*x*_S_5_Br.

## Conclusion

The origin of higher conductivities in Si-substituted Li_6_PS_5_Br (Li_6+*x*_P_1−*x*_Si_*x*_S_5_Br) is investigated using *ab initio* molecular dynamics. The simulations show that the excess Li concentration, originating from the charge balance in the Si^4+^ doped structures, resides on the T4 (Wyckoff 16e) sites. Rietveld refinement of neutron diffraction patterns confirms the presence of Li in the T4 position. A radial distribution function analysis reveals that this partial occupancy distributes Li more uniformly between the T5 and T4 positions, effectively flattening the energy landscape and increasing Li mobility through the T4 site. This increase is confirmed by detecting increasing jump rate statistics for this jump path. Because the T4 sites reside between two bordering Li cages, the intercage diffusion increases as a function of the Si concentration. As intercage jumps are directly related to macroscopic diffusion and ionic conductivity, a corresponding increase in Li-ion conductivity is demonstrated in Arrhenius plots for higher doping concentration. However, increasing ionic conductivity for higher doping concentrations is compromised by a higher reduction potential, reducing the electrochemical stability for increased Si doping.

## Author contributions

TS, AL, DD, and AV performed DFT simulations. TS, AL, and AV analyzed the results. AG analyzed neutron diffraction data. TS, MW, AG, and AV designed the project. All authors wrote, contributed to, and discussed the final manuscript.

## Conflicts of interest

The authors declare no competing financial interests.

## Supplementary Material

MA-005-D3MA01042B-s001
