# Supplementary material for: Understanding the role of aliovalent cation substitution on the li-ion diffusion mechanism in Li6+xP1−xSixS5Br argyrodites
Source: Mater Adv. 2024 Jan 15;5(5):1952–9. doi: 10.1039/d3ma01042b (PMC10911230; doi:10.1039/d3ma01042b)
Supplement: MA-005-D3MA01042B-s001 [file MA-005-D3MA01042B-s001.pdf]

Electronic Supplementary Material (ESI) for Materials Advances.
This journal is © The Royal Society of Chemistry 2024

Understanding the Role of Aliovalent Cation Substitution on the Li-ion Diffusion Mechanism in $Li_{6+x}P_{1-x}Si_xS_5Br$ Argyrodites

Tammo K. Schwietert[a], Ajay Gautam[a], Anastasia K. Lavrinenko[a], David Drost[a], Theodosios Famprikis[a], Marnix Wagemaker[a*] and Alexandros Vasileiadis[a,*]

[a]*Storage of Electrochemical Energy, Department of Radiation Science and Technology, Faculty of Applied Sciences, Delft University of Technology, Mekelweg 15, 2929JB, Delft, The Netherlands*

*Corresponding authors: m.wagemaker@tudelft.nl, a.vasileiadis@tudelft.nl

Supplementary information

A.

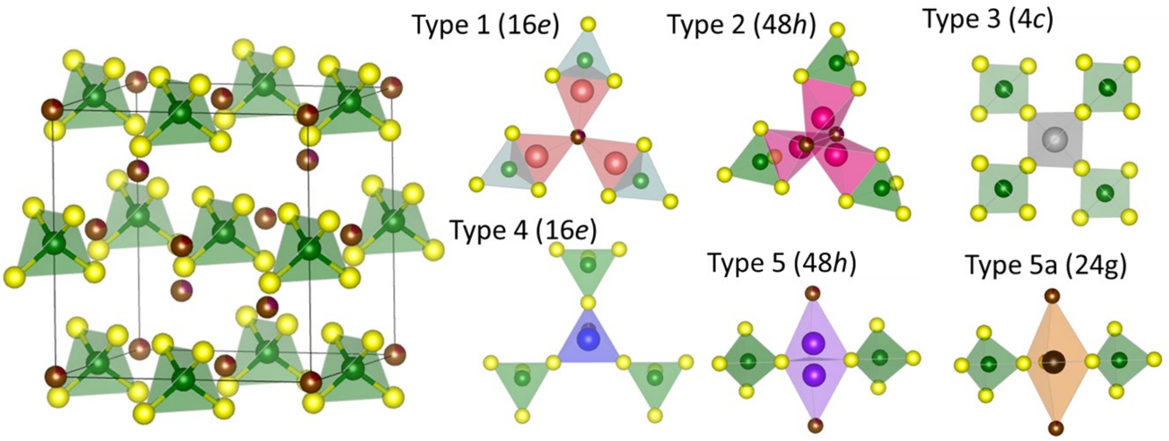

***Figure S1**: Crystal structure of Li_6PS_5X (X = Cl, Br, or I), where X^- positioned on the Wyckoff 4a site and S^{2-} on the Wyckoff 4c site. The argyrodite framework highlights S^{2-} anions that are tetrahedrally close-packed on Wyckoff positions (4d, 16e), and determines 136 voids in the unit cell. Four are occupied by P^{5+} at the 4b site, forming the $PS_4{}^{3-}$. Panels depicting the trigonally coordinated type 5a site and the five types of tetrahedral interstitial sites (Types T1-T5) that could possibly host lithium, as proposed in reference 1.*

B.

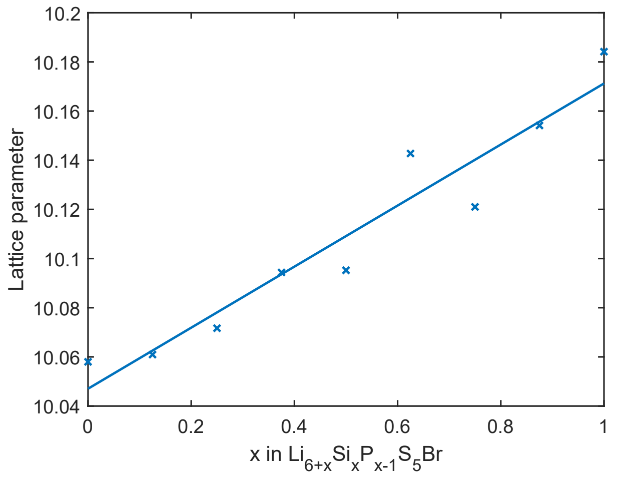

Figure S2 *Lattice parameters determined after a DFT relaxation for different values of x in the $Li_{6+x}Si_xP_{1-x}S_5Br$ structure.*

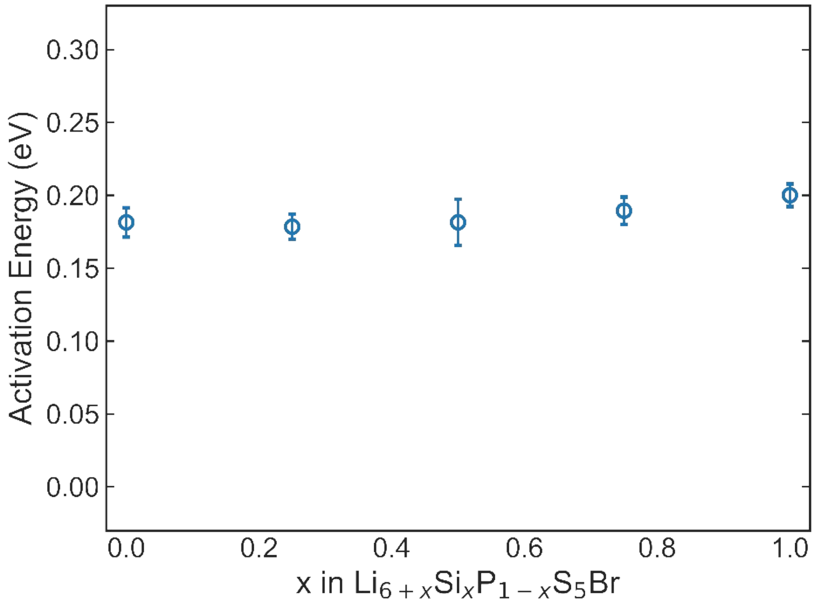

Figure S3 *Activation energies of conductivities calculated for $Li_{6+x}Si_xP_{1-x}S_5Br$.*

C.

Table S1 *Rietveld refinement of pristine* Li_6PS_5Br *at room temperature. Lattice parameters, fractional atomic coordinates, isotropic atomic displacement, and site occupancies are refined.*

**Li_6PS_5Br**	**Wyckoff**	**X**	**Y**	**Z**	**Occ.**	**Biso**
**F-43m**	48h T5	0.3036(11)	0.0231(12)	0.696(1)	0.30(2)	2.2(4)
**a = 9.9836 (2) Å**	48h T2	0.296(8)	0.087(6)	0.587(6)	0.13(2)	10(2)
	24g T5a	0.25	0.013(7)	0.75	0.14(6)	2.2(4)
	4b P	0	0	0.5	1.0	1.45(7)
	16e S	0.1185(4)	-0.1185(4)	0.6185(4)	1.000	2.03(7)
	4a S	0.0	0.0	0.0	0.10(2)	1.60(9)
	4c S	0.25	0.25	0.75	0.90(2)	2.92(11)
	4a Br	0.0	0.0	0.0	0.90(2)	1.60(9)
	4c Br	0.25	0.25	0.75	0.1(2)	2.92(11)

Table S2 *Rietveld refinement of pristine* $Li_{6.125}P_{0.875}Si_{0.125}S_5Br$ *at room temperature. Lattice parameters, fractional atomic coordinates, isotropic atomic displacement, and site occupancies are refined.*

**$Li_{6.125}P_{0.875}Si_{0.125}S_5Br$**	**Wyckoff**	**X**	**Y**	**Z**	**Occ.**	**Biso**
**F-43m**	48h T5	0.3044(5)	0.0230(2)	0.695(2)	0.45(2)	3.87(2)
**a = 10.0121(1) Å**	48h T2	0.278(2)	0.413(1)	0.913(1)	0.025(1)	8(2)
	16e	0.153(2)	0.153(2)	0.153(2)	0. 08(1)	1.1(2)
	4b P	0	0	0.5	0.875(2)	3.26(9)
	4b Si	0	0	0.5	0.125 (2)	3.26(9)
	16e S	0.1164(2)	-0.1164(2)	0.6164(2)	1.000	3.72 (5)
	4a S	0.0	0.0	0.0	0.18(2)	1.41(2)
	4c S	0.25	0.25	0.75	0.82(2)	2.76(1)
	4a Br	0.0	0.0	0.0	0.82(2)	1.41(2)
	4c Br	0.25	0.25	0.75	0.18(2)	2.76(1)

Table S2 *Normalized occupations and comparison between experiments and computations.*

site	multi	x	T	Occ.	maxLi	normalized_Occup	cumulative__Occup
T5	48	0.125	MD 600K (all)	0.1828	24.5	35.82	35.823
T2	48	0.125	MD 600K (all)	0.0693	24.5	13.57	49.398
T4	16	0.125	MD 600K (all)	0.0435	24.5	2.84	52.238
T5	48	0.125	MD 600K (solo)	0.3423	24.5	67.06	67.056
T2	48	0.125	MD 600K (solo)	0.1204	24.5	23.6	90.652
T4	16	0.125	MD 600K (solo)	0.1070	24.5	6.989	**97.641**
T5	48	0.125	Experiment	0.454	24.5	88.95	88.947
T2	48	0.125	Experiment	0.025	24.5	4.898	93.845
T4	16	0.125	Experiment	0.077	24.5	5.029	**98.873**

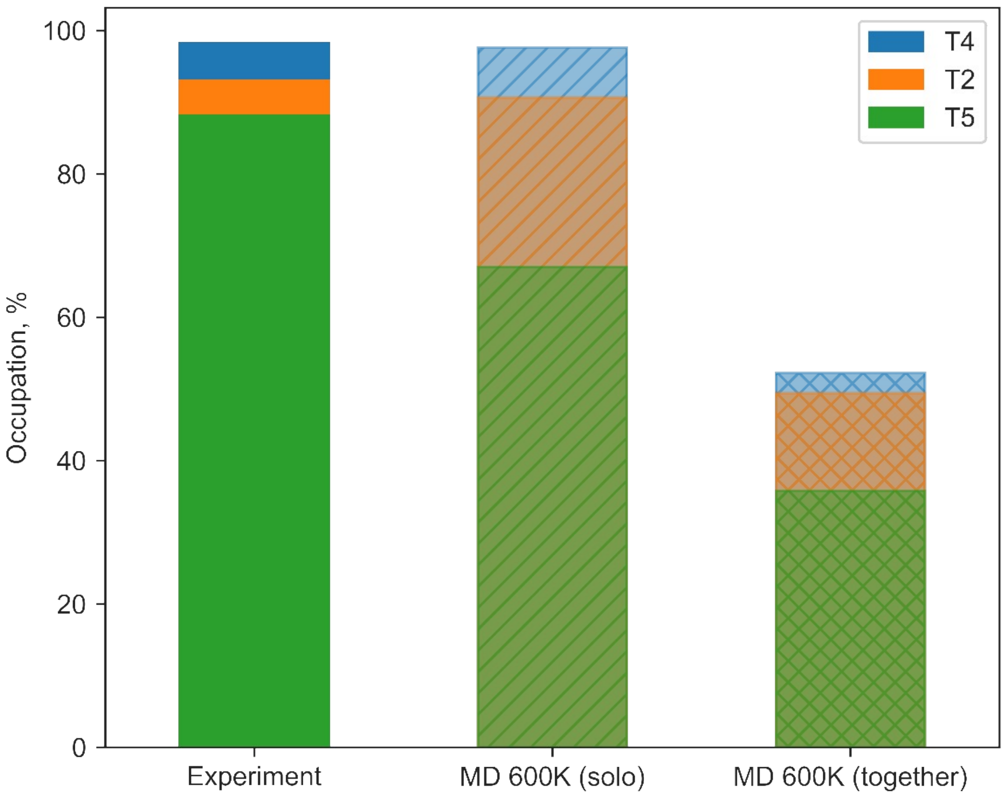

Figure S5 *Comparison of normalized occupations between experimental data, molecular dynamics when interstitials are probed individually, and molecular dynamics when interstitials are probed all together for* $Li_{6+x}Si_xP_{1-x}S_5Br$ *configuration.*

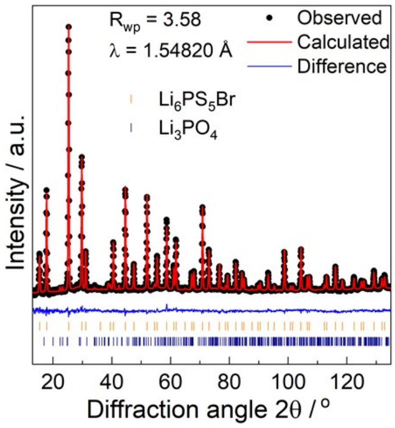

Figure S6 *(a) Rietveld refinement analysis of neutron diffraction data of* Li_6PS_5Br*, showing a small fraction (0.6 wt%) of the impurity phase* Li_3PO_4*.*

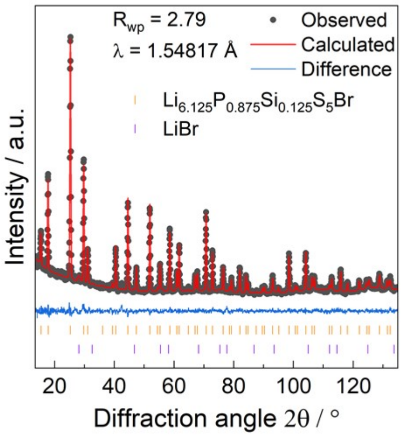

Figure S7 *(a) Rietveld refinement analysis of neutron diffraction data of* $Li_{6.125}P_{0.875}Si_{0.125}S_5Br$*, showing a small fraction (2.3 wt%) of the impurity phase LiBr.*

Bibliography

1. Kong, S. T.; Deiseroth, H. J.; Reiner, C.; Gün, Ö.; Neumann, E.; Ritter, C.; Zahn, D. Lithium Argyrodites with Phosphorus and Arsenic: Order and Disorder of Lithium Atoms, Crystal Chemistry, and Phase Transitions. Chemistry - A European Journal **2010**, 16, 2198–2206.
